# How have ART treatment programmes changed the patterns of excess mortality in people living with HIV? Estimates from four countries in East and Southern Africa

**DOI:** 10.3402/gha.v7.22789

**Published:** 2014-04-22

**Authors:** Emma Slaymaker, Jim Todd, Milly Marston, Clara Calvert, Denna Michael, Jessica Nakiyingi-Miiro, Amelia Crampin, Tom Lutalo, Kobus Herbst, Basia Zaba

**Affiliations:** 1 Department of Population Health, London School of Hygiene and Tropical Medicine, London, UK; 2 TAZAMA Project, National Institute for Medical Research, Mwanza, Tanzania; 3 Department of Infectious Disease Epidemiology, London School of Hygiene and Tropical Medicine, London, UK; 4 Medical Research Council/Uganda Virus Research Institute (MRC/UVRI), Research Unit on AIDS, Entebbe, Uganda; 5 Karonga Prevention Study, Chilumba, Malawi; 6 Rakai Health Sciences Program, Uganda Virus Research Institute, Entebbe, Uganda; 7 The Africa Centre for Health and Population Studies, University of KwaZulu-Natal (UKZN), Somkhele, South Africa

**Keywords:** HIV, sub-Saharan Africa, mortality, ALPHA network, antiretroviral therapy

## Abstract

**Background:**

Substantial falls in the mortality of people living with HIV (PLWH) have been observed since the introduction of antiretroviral therapy (ART) in sub-Saharan Africa. However, access and uptake of ART have been variable in many countries. We report the excess deaths observed in PLWH before and after the introduction of ART. We use data from five longitudinal studies in Malawi, South Africa, Tanzania, and Uganda, members of the network for Analysing Longitudinal Population-based HIV/AIDS data on Africa (ALPHA).

**Methods:**

Individual data from five demographic surveillance sites that conduct HIV testing were used to estimate mortality attributable to HIV, calculated as the difference between the mortality rates in PLWH and HIV-negative people. Excess deaths in PLWH were standardized for age and sex differences and summarized over periods before and after ART became generally available. An exponential regression model was used to explore differences in the impact of ART over the different sites.

**Results:**

127,585 adults across the five sites contributed a total of 487,242 person years. Before the introduction of ART, HIV-attributable mortality ranged from 45 to 88 deaths per 1,000 person years. Following ART availability, this reduced to 14–46 deaths per 1,000 person years. Exponential regression modeling showed a reduction of more than 50% (HR =0.43, 95% CI: 0.32–0.58), compared to the period before ART was available, in mortality at ages 15–54 across all five sites.

**Discussion:**

Excess mortality in adults living with HIV has reduced by over 50% in five communities in sub-Saharan Africa since the advent of ART. However, mortality rates in adults living with HIV are still 10 times higher than in HIV-negative people, indicating that substantial improvements can be made to reduce mortality further. This analysis shows differences in the impact across the sites, and contrasts with developed countries where mortality among PLWH on ART can be similar to that of the general population. Further research is urgently needed to establish why the different impacts on mortality were observed and how the care and treatment programmes in these countries can be more effective in reducing mortality further.

Prior to the advent of antiretroviral therapy (ART), observed mortality rates among adults living with HIV from population-based studies were 10–15 times higher than in HIV-negative adults (1–4). This indicates that 90–95% of deaths in people living with HIV (PLWH) were excess deaths due to HIV infection and were therefore attributable to HIV. The proportion of deaths in PLWH that are due to HIV depends on the background mortality in the population (5).

Clinic-based data have shown substantial falls in mortality since the introduction of ART, but mortality among PLWH is still considerably higher than among individuals who are HIV-negative. ART programmes have been shown to have substantial impacts on all-cause and AIDS mortality in sub-Saharan African countries (6–10) despite the poorer survival documented among people receiving treatment in low-income countries compared to those in high-income countries (11).

In developing countries, there are many barriers to effective ART provision. On an individual level, PLWH who have not received HIV testing and counselling (HTC), or have not accessed ART clinics, do not receive treatment (12). Within ART treatment programmes, there may be inadequate treatment of other infections and limited social support. Monitoring of immunological recovery, adherence, and drug resistance is difficult in many settings, resulting in suboptimal delivery of ART. Factors that influence wider health, such as food security and access to health services, also influence the uptake and effectiveness of ART. All these factors can limit the impact of ART on mortality (13).

Therefore, in sub-Saharan African countries, where the relatively recent ART rollout has been shown to have some impact on mortality, further reductions in HIV-positive mortality may be possible, and this raises the question of the number of excess deaths in PLWH, even when treatment programmes are in place. In order to estimate the extent of the mortality decline among PLWH since the introduction of ART, and to estimate what further reductions may be possible, we need data on the HIV serostatus of the general population, which is not widely available.

This article reports results from studies in Malawi (the Karonga Prevention Study, London School of Hygiene and Tropical Medicine), South Africa (the uMkhanyakude cohort, Africa Centre for Health and Population Studies), Tanzania (the Kisesa cohort, National Institute for Medical Research), and Uganda (the Masaka-Kyamulibwa general population cohort, Medical Research Council/Uganda Virus Research Institute; and the Rakai Health Sciences Program), which form part of the network for Analysing Longitudinal Population-based HIV/AIDS data on Africa (ALPHA), which brings together the sites in sub-Saharan Africa that collect longitudinal, population-based demographic data alongside HIV serostatus. These sites provide unique data on mortality pre- and post-ART. Demographic data are available for a long period (starting from 1989), and from five communities in four countries with different HIV epidemic settings. Data on HIV serostatus are available from different time points in some sites; these dates are given in Table 1. These data permit estimation of mortality rates in PLWH and HIV-negative people and the extent of excess deaths in PLWH. Detailed analyses of mortality in each site have been conducted (14–16).

**Table 1:** Dates for the introduction of ART, the full rollout of ART, and the start of demographic surveillance and HIV serosurveys

			Start dates for ART phases		
			
Site	Start of demographic data collection	Start of HIV serosurveys	Pre-ART phase	ART introductory phase	Post-ART phase
Karonga	August 2002	October 2005	July 2000	July 2005	October 2006
Kisesa	May 1994	August 1994	January 2000	March 2005	October 2008
Masaka	November 1989	November 1989	January 1999	January 2004	January 2005
Rakai	April 1999	April 1999	June 1999	June 2004	June 2006
uMkhanyakude	January 2000	October 2002	January 1999	January 2005	January 2007

The study sites are in predominantly rural areas. HIV prevalence is highest in uMkhanyakude [29% among 15–49 year olds in 2011 (17)], where the epidemic started later and reached a higher level than in the other sites and where ART is helping maintain high prevalence by averting deaths among PLWH. Prevalence in the countries with more established epidemics is stable; it is substantially lower in Karonga, Kisesa, and Masaka (~6%) and intermediate in Rakai. ART is provided through national treatment programmes, although Masaka delivers ART through a study clinic rather than a government health facility.

In this article, we compare the number of excess deaths in PLWH in three different periods: during the 5 years before the introduction of ART, at the time during which each country's ART programme was established, and the 5 years after ART became widely available in each country. We use an exponential regression model to assess how the death rate among PLWH has changed relative to that among HIV-negative people during these same time periods.

## Methods

### Data collection methods

This article uses data from 5 of the 10 sites that make up the ALPHA network. The sites which contributed data are Karonga (Malawi), Kisesa (Tanzania), Masaka (Uganda), Rakai Health Sciences Program (Uganda), and the Africa Centre (uMkhanyakude, South Africa), and their fieldwork methods have been described elsewhere (18–24). Each site runs a demographic surveillance system (DSS) with linked HIV serostatus information on adult residents. Four sites have demographic and HIV status data spanning the three periods of interest surrounding the introduction of ART. The fifth (Karonga) is included for the purposes of cross-site comparisons.

Each study site extracted, from their study databases, information describing time spent resident in the study area and including dates of exit from the study, due to death or out-migration. They also extracted all information on the HIV status of each study participant. Data were prepared in a standard format, and data from all sites were pooled in February 2013.

### Data definitions

HIV test information was merged with the residency episodes. The HIV status during each part of a residency episode was derived from the test results at specific time points. To ensure consistency between the classification of deaths and of exposure time, person years were classified by HIV status as shown in Table 2. All deaths occurring in persons with a documented positive HIV test result arising from an ante-mortem study test were classified as HIV-related, and these composed the numerator for the HIV-positive mortality rate. Deaths amongst persons who had recently been tested in the study and were found to be HIV-negative formed the numerator for the negative mortality rate.

**Table 2:** Classification of deaths and exposure time by HIV status

HIV-negative	HIV-positive	HIV status unknown
Between successive negative tests	Following any positive test	Never tested
Up to X years following a negative test with no other subsequent test		Not yet tested (i.e. Before first test, whether or not that first test was positive or negative)
In a seroconversion interval less than X years duration (negative test followed by positive test)		More than X years following the last negative test

X was based on observed incidence rates in the sites, and the values used were 2 years in uMkhanyakude, 3 years in Rakai, and 5 years elsewhere.

Data on the introduction of ART in each country were used to identify three periods: before the introduction of ART (i.e. no treatment available); a rollout phase immediately following the introduction of ART, when ART was assumed to be only partially available; and, subsequently, a phase when ART was widely available (Table 1). In these observational study sites, ART was introduced when national treatment programmes started to provide services. These periods therefore do not precisely align with the demographic and serosurvey activities in all sites, and thus data are not available for certain periods in some sites.

### Statistical methods

Each individual contributed exposure time during their residence episodes in the DSS, which is from entry into the DSS until exit due to migration, death, or censoring at the time of the latest survey (or refusal to continue, where relevant). Returning migrants, individuals who re-entered the study following out-migration, contributed person years only from the time they were resident in the study site (i.e. between first entry and first exit and between each subsequent entry and exit, as applicable).

To calculate mortality attributable to HIV, age-specific mortality rates for HIV-negative people were applied to the HIV-positive person years to calculate the number of deaths expected in the HIV-positive group, had that group experienced the mortality rates of the HIV-negative individuals. The number of excess deaths were classified as HIV attributable, and they were summarized over the time periods and over sex and age groups.

The age and sex composition of the populations differed between sites and over the ART phases. Mortality rates were standardized to allow comparison between sites and across ART phases. The standard population was the total number of person years contributed from all sites in all ART phases.

A series of exponential regression models were fitted, stratified by 5-year age group, to compare the mortality rate among PLWH to that among HIV-negative individuals. The baseline group for comparison was HIV-negative individuals in the later period when ART was widely available. Mortality amongst HIV-negative individuals in the two earlier phases, and mortality among PLWH in each ART phase, were compared by fitting an interaction term in the model between HIV status and ART phase. Sex and study site were included in the model as fixed effects, and the standard errors were adjusted for clustering by study site. By analyzing the data in this way, the additional mortality due to HIV can be captured, since HIV-negative individuals experienced a baseline level of mortality. Data from Karonga were not included in the model since they contributed data only to the post-ART phase. Those with unknown HIV status were included in this model to account for differences over time in the ascertainment of HIV status. These differences are most apparent in the two sites with the most recent introduction of HIV testing (Karonga and uMkhanyakude).

## Results

The five study sites provided data on 244,769 adults aged 15 and older, who contributed a total of 1,149,484 person years. This analysis is concerned with the mortality of the 127,585 adults age 15 years and above who have known HIV status and who contributed 487,242 person years. Table 3 shows for each site the number of subjects, person years, the mortality rate, and the period prevalence of HIV. The period prevalence of HIV was stable in Kisesa and Rakai, showed a moderate increase in Masaka, and showed a steep increase in uMkhanyakude. The proportion of all deaths that were in known HIV positive individuals declined over the three periods in all four sites where this comparison was possible. All-cause mortality ranged from 7 to 19 deaths per 1,000 person years.

**Table 3:** Number of subjects and person years contributed to the analysis, mortality rate and period prevalence of HIV amongst persons with known HIV status, and the proportion of all deaths that were of individuals known to be HIV positive

ART phase	Number of subjects	Person years	Deaths	Mortality rate	Period prevalence	HIV-positive deaths (% of total)
Karonga						
0–5 years before ART	*	*	*	*	*	*
ART introduction	*	*	*	*	*	*
ART available	18,159	46,134	316	6.8	7.4	25.9
Kisesa						
0–5 years before ART	13,153	34,491	412	11.9	5.2	31.8
ART introduction	12,014	27,234	317	11.6	5.0	25.6
ART available	11,303	21,639	185	8.5	5.6	25.4
Masaka						
0–5 years before ART	10,873	31,336	500	16.0	5.6	42.8
ART introduction	8,275	7,268	107	14.7	5.8	34.6
ART available	12,610	39,867	436	10.9	6.6	25.9
Rakai						
0–5 years before ART	22,845	66,327	616	9.3	12.8	79.7
ART introduction	20,814	31,426	243	7.7	12.7	77.8
ART available	28,289	72,732	588	8.1	12.6	67.0
uMkhanyakude						
0–5 years before ART	12,470	10,177	177	17.4	20.6	82.5
ART introduction	18,458	25,625	405	15.8	22.3	75.8
ART available	29,346	72,987	1,349	18.5	27.1	55.6
All sites pooled						
0–5 years before ART	59,341	142,330	1,705	12.0	9.9	57.6
ART introduction	59,561	91,553	1,072	11.7	12.5	57.3
ART available	99,707	253,359	2,874	11.3	14.3	48.2

* Estimate not available.

The standardized mortality rates given in Table 4 show the mortality rates for HIV-negative individuals and PLWH and the excess mortality rate among PLWH, by site, ART phase, and sex. These rates are standardized to adjust for differences in the age composition between sites and over ART phases. In all groups, the excess mortality rate among PLWH was almost as high as the total mortality rate for PLWH and much higher than the mortality rate among HIV-negative individuals. The HIV-positive mortality rate varied considerably between the sites, and the excess mortality rate reflected that. With the exception of men in Rakai, declines are apparent in both the HIV positive overall and excess mortality, comparing post-ART rollout with the time before ART was available.

**Table 4:** Age-standardized mortality rates (per 1,000 person years) by site, sex, HIV status, and ART phase: rates for HIV-negative people and PLWH, the excess mortality rate among PLWH (rate difference), and the standardized mortality ratio (SMR)

	Men				Women			
		
Site and ART phase	HIV-negative mortality rate	HIV-positive mortality rate	Attributable	SMR	HIV-negative mortality rate	HIV-positive mortality rate	Attributable	SMR
Karonga								
0–5 years before ART	–	–	–		–	–	–	
ART introduction	–	–	–		–	–	–	
ART available	2.5	24.4	22.0	10	1.7	15.0	13.7	8.8
Kisesa								
0–5 years before ART	3.6	55.2	51.6	15.3	4.2	52.4	48.7	12.5
ART introduction	5.2	47.1	42.2	9.1	3.2	39.8	36.9	12.4
ART available	3.2	33.8	30.8	10.6	1.8	19.6	18.3	10.9
Masaka								
0–5 years before ART	3.9	88.0	84.8	22.6	3.2	91.5	88.3	28.6
ART introduction	2.8	77.4	75.4	27.6	3.5	60.5	58.0	17.3
ART available	3.8	43.6	39.9	11.5	2.3	30.3	28.3	13.2
Rakai								
0–5 years before ART	2.3	42.5	40.6	17.7	1.7	45.7	43.9	25.8
ART introduction	2.2	31.1	28.9	13.1	1.0	32.8	31.8	31.8
ART available	3.2	49.4	46.2	14.4	2.5	28.3	25.8	10.3
uMkhanyakude								
0–5 years before ART	7.6	63.4	56.2	8.3	1.8	56.7	54.9	31.5
ART introduction	8.3	47.4	39.5	5.7	2.1	38.3	36.1	18.2
ART available	5.7	43.2	37.5	7.6	1.6	22.6	21.1	14.1

**Fig. 1:**
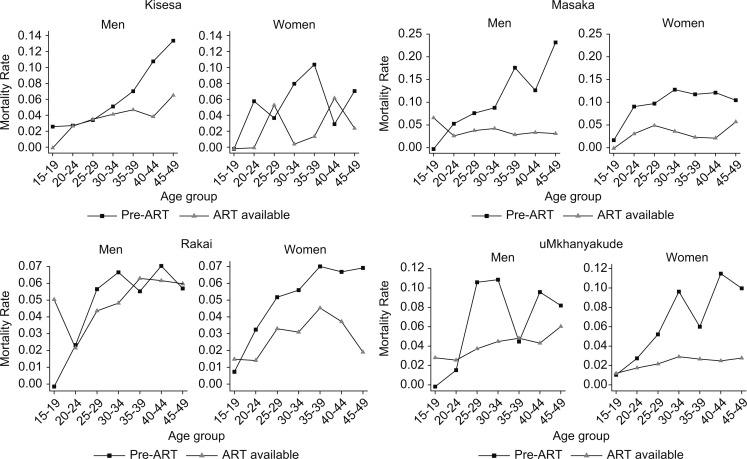
Estimated excess mortality rate among PLWH by age, sex, and ART phase.

Before the advent of ART, the excess mortality rate among PLWH was higher for men than for women. For men and women, excess mortality was highest in Masaka and lowest in Rakai. When ART became available, the mortality rates fell; the extent of the decrease varied between the site and across the sites by age group. Figure 1 shows the excess mortality rates by age, sex, and ART phase for Kisesa, Masaka, Rakai, and uMkhanyakude. Excess mortality rates for Karonga can only be estimated for the post-ART phase and hence are not shown in Fig. 1. In general, the most pronounced falls were in the older age groups, whereas among 15–19-year-old men in Masaka, Rakai, and uMkhanyakude and 15–19-year-old women in Rakai, excess mortality rates increased slightly.

**Fig. 2:**
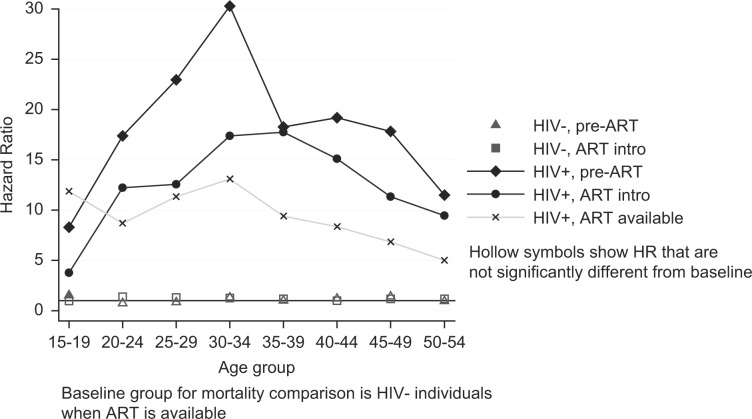
Hazard ratios from an age-stratified exponential regression model comparing the mortality of PLWH to that of HIV-negative individuals, by ART availability. Estimates are controlled for sex and study site; these results are given in Table 5.

The impact of ART availability is shown in Fig. 2 and Table 5, which contain results from an age-stratified exponential regression model comparing the mortality of PLWH and HIV-negative individuals.

**Table 5:** Hazard ratios and 95% confidence intervals from an age-stratified exponential model comparing the mortality of PLWH and HIV-negative individuals by ART phase

ART phase and HIV status	15–19	20–24	25–29	30–34	35–39	40–44	45–49	50–54
Pre-ART HIV−	1.53 (1.14–2.05)	0.75 (0.44–1.25)	0.83 (0.69–1.00)	1.31 (0.48–3.54)	1 (0.57–1.74)	1.2 (0.51–2.82)	1.4 (0.70–2.84)	0.94 (0.54–1.62)
Pre-ART HIV+	8.29 (6.21–11.0)	17.38 (13.66–22.1)	22.96 (18.32–28.7)	30.3 (18.17–50.5)	18.27 (10.04–33.2)	19.2 (15.96–23.1)	17.83 (11.87–26.7)	11.49 (5.73–23.0)
Pre-ART HIV?	2.07 (1.55–2.76)	4.22 (2.85–6.25)	7.11 (4.63–10.9)	9.45 (6.51–13.7)	5.85 (4.37–7.83)	4.97 (3.80–6.50)	3.66 (3.09–4.33)	1.9 (1.74–2.07)
ART intro HIV−	0.97 (0.59–1.59)	1.39 (0.78–2.46)	1.3 (0.80–2.10)	1.26 (0.45–3.52)	1.18 (0.33–4.22)	1 (0.57–1.74)	1.18 (0.83–1.67)	1.17 (0.72–1.92)
ART intro HIV+	3.76 (1.79–7.90)	12.22 (9.27–16.1)	12.57 (9.31–16.9)	17.38 (12.22–24.7)	17.76 (15.81–19.9)	15.1 (14.49–15.7)	11.33 (8.76–14.6)	9.45 (5.20–17.1)
ART intro HIV?	2.01 (1.69–2.38)	3.11 (1.62–6.00)	6.56 (4.45–9.67)	8.79 (4.92–15.6)	4.72 (3.15–7.10)	4.19 (2.82–6.23)	2.75 (2.04–3.70)	2.18 (1.77–2.67)
Post-ART HIV−	1	1	1	1	1	1	1	1
Post-ART HIV+	11.85 (8.81–15.9)	8.7 (7.02–10.7)	11.33 (7.95–16.1)	13.09 (10.99–15.5)	9.4 (7.89–11.2)	8.35 (5.33–13.0)	6.84 (5.14–9.11)	5 (4.23–5.91)
Post-ART HIV?	2.26 (1.34–3.79)	3.72 (2.27–6.08)	6.34 (3.48–11.5)	7.93 (5.33–11.8)	5.03 (4.13–6.13)	4.07 (2.58–6.42)	2.87 (2.13–3.87)	2.07 (1.33–3.23)
Sex								
Men	1	1	1	1	1	1	1	1
Women	1.02 (0.93–1.11)	1.32 (1.12–1.56)	1.06 (0.98–1.16)	0.9 (0.76–1.06)	0.75 (0.49–1.15)	0.59 (0.44–0.79)	0.57 (0.45–0.72)	0.53 (0.42–0.68)
Study site								
Kisesa	1	1	1	1	1	1	1	1
Masaka	0.98 (0.88–1.09)	1.92 (1.66–2.21)	1.91 (1.75–2.10)	2.07 (1.85–2.30)	1.56 (1.42–1.71)	1.19 (1.06–1.34)	1.29 (1.18–1.40)	0.94 (0.92–0.96)
Rakai	0.94 (0.88–1.01)	1.35 (1.26–1.44)	1.74 (1.61–1.88)	1.87 (1.74–2.00)	1.62 (1.52–1.73)	1.12 (1.03–1.23)	0.99 (0.91–1.07)	1.18 (1.10–1.26)
uMkhanyakude	0.88 (0.79–0.99)	1.5 (1.39–1.62)	1.97 (1.84–2.11)	2.36 (2.10–2.64)	1.79 (1.54–2.08)	1.64 (1.40–1.92)	1.76 (1.56–1.99)	1.66 (1.47–1.87)

These results are from the same model as the estimates presented in Fig. 2.

Figure 2 shows that the mortality of HIV-negative individuals changed little between the three ART periods; the only significant difference was that the mortality of 15–19 year olds was slightly higher in the earliest period (pre-ART). Within each age group, the mortality of PLWH remained much higher than that of HIV-negative people, even following the introduction of ART. Among 30–34 year olds, the risk of mortality for a PLWH before the introduction of ART was 30 times higher (95% CI: 18–51) than that of an HIV-negative person. Following the introduction of ART, the relative mortality hazard had more than halved, but it remained 13 times higher (95% CI: 11–16) than that of HIV-negative individuals. In the introductory phase of ART, the mortality reduction was greatest at younger ages (those younger than 35, the peak group for mortality). After ART was fully established, mortality risk declined in older age groups, which meant that the excess mortality risk among PLWH was more uniformly distributed across the age range, with a hazard ratio (HR) of 12 (95% CI: 9–16) in 15–19 year olds, 13 (95% CI: 11–16) in 30–34 year olds, and 5 (95% CI: 4–6) among 50–54 year olds.

Differences are apparent between the study sites at different ages, reflecting the age-specific incidence pattern. Masaka, Rakai, and uMkhanyakude's mortality was higher than Kisesa's between the ages of 20 and 44. Mortality in uMkhanyakude remained higher than that of the other sites in the older age groups as well. There were also differences between the sexes at different ages: women's mortality hazard was higher than men's in the 20- to 24-year-old age group; and, from the age of 30, the direction of this association reversed and men's mortality risk was higher than women's such that by their late 40s, women's risk of dying was 60% that of men.

The changes between the ART phases are evident with respect to a fixed comparison group. The models do not show whether, between the ART phases, the mortality changes among the PLWH are likely to be true. Separate analysis, not shown, comparing the mortality changes between the phases among PLWH using stratified exponential regression confirms that the changes observed are highly unlikely to be due to chance. There was a significant difference between the mortality hazards for each ART phase. For PLWH in their 20s and older, the mortality had changed significantly between the pre- and post-ART phase. No difference in mortality was apparent for teenagers living with HIV.

## Discussion

Assessment of the mortality attributable to HIV requires information on HIV serostatus and cause of death. In countries where there is no adequate system of vital registration, DSSs provide important estimates of levels and trends in mortality. The members of the ALPHA network, whose data are used here, combine the information from demographic surveillance with information on HIV serostatus from serological surveillance.

From these five sites, we have HIV status data on a large proportion of five distinct populations covering areas of very different HIV prevalence. These data can be used to look at trends in HIV-positive mortality rates and the excess mortality in PLWH. Excess mortality rates are obtained from the difference between the mortality rate of the PLWH and the mortality rate of the HIV-negative individuals. In all sites, at least 90% of the deaths among PLWH were attributable to HIV: they were in excess of the deaths that would be expected given background mortality rates among the HIV-negative population.

In the Karonga district of Malawi, the fraction of mortality in the population attributable to HIV has decreased since the advent of ART (25). In this analysis, Karonga is the site with the lowest overall and excess mortality among PLWH.

The reasons for this excess are unknown. If HIV infection remains undiagnosed, the individual cannot access treatment and lower their risk of dying. Suboptimal treatment and associated care may not be enough to prevent death. Late presentation may make it unlikely that treatment will succeed. Data on individual experience of HIV diagnosis, treatment, and care will help to explain why mortality remains high. These data are difficult to collect from the general population and insufficient data were available at the point of writing, but future analyses are planned.

Two large international trials showed that among PLWH who achieve adequate viral suppression and CD4^+^ T-cell counts of at least 500 cells/ml, the mortality rate is the same as that experienced by the general population (26). A European study reached a similar conclusion, finding in a clinical cohort that the standardized mortality ratio (SMR) was 4.2, but in the subset of individuals with a CD4 count higher than 500/mm^3^, the SMR was 1.5 (27). The International Epidemiologic Databases to Evaluate AIDS (IeDEA) consortium carried out an analysis of PLWH receiving ART in Côte d'Ivoire, Malawi, South Africa, and Zimbabwe; they found that, among people who started ART with a CD4 count over 200 and at World Health Organization (WHO) clinical stage I or II, the mortality rate was very similar to that of the general population (28). However, the same study estimated an overall HIV-attributable mortality rate of 69.5 per 1,000 person-years among the entire group receiving treatment and an SMR of 18.7. These estimates are higher than those reported in this article, and one reason could be that the follow-up ended in 2007 when ART programmes were still quite new. Analysis of data from 13 clinical cohort studies with follow-up between 1996 and 2006 found a crude death rate of 12.1 per 1,000 person years among PLWH receiving ART (29). Another analysis of nine cohorts found crude mortality rates among PLWH on ART that ranged from 2.9 to 22.9 per 1,000 person years, with a pooled crude rate of 9.5 per 1,000 (30).

However, the people identified as living with HIV in the ALPHA studies are from population-based cohorts, which include those who are on ART, those who have not yet started ART, and those who are undiagnosed. Unlike the clinical cohort studies, participants in the observational ALPHA cohorts may remain undiagnosed or untreated at an advanced stage of infection, even once ART is available. This might explain the higher mortality rate among PLWH in the ALPHA sites compared to the rates estimated from studies in Europe and North America. The HIV-positive mortality rates from some of the ALPHA sites lie within the range from the clinical cohorts, even though ART coverage does not reach 100% in any site. This may be because the ALPHA sites regularly test all individuals who participate in the studies, and many of those subsequently identified as living with HIV will be at the early stages of HIV infection and initially experience little excess mortality. Individuals like this are likely to be underrepresented in a clinical cohort. This may also explain why the ALPHA estimates are lower than those from the IeDEA study (28).

A strength of these results is that the estimation of background mortality is directly measured – available from the same population and at the same time – whereas most previous studies have relied on national estimates. The validity of these findings depends on the assumption that the ascertainment of HIV status is essentially complete, with the HIV status and stage of infection among those who have not been tested assumed to be the same as among those whose status is known. If this is not the case, then the true difference in mortality between PLWH and HIV-negative individuals may be different from that estimated. The proportion of individuals (aged 15+) in the DSS for whom HIV status had never been measured, by the end of the last phase of follow-up, ranged from 16% in Masaka (the longest running study) to 60% in uMkhanyakude (the site with the shortest history of HIV testing).

In this article, we have not used data on cause of death to distinguish AIDS mortality from other causes of death among PLWH, which is another way of estimating mortality attributable to HIV. Population-based cause-of-death information is not routinely available in these countries. A variable proportion of recorded deaths in ALPHA sites have had a verbal autopsy (VA) done. VA data can be used to assign a probable cause of death for people dying outside hospital settings either by physician review or through the use of software. We have not used VA data in this article because the differences in VA coverage between the sites, and over time, are sufficient to undermine our ability to make comparisons between sites and over time.

VA data have been used to show that HIV-associated deaths are a major contributor to adult mortality in different sub-Saharan African populations (25, 31). VA data have also been used to show the share of AIDS deaths in the population as a whole, which depends on the prevalence of HIV in the population as well as the background mortality in HIV-negative individuals.

The Global Burden of Disease study of 2010 derived cause-specific mortality estimates by modeling data from a variety of sources. They estimated that the proportion of all deaths attributable to HIV/AIDS was highest in South Africa (41.1%) and lower in Malawi (23.6%), Tanzania (21.2%), and Uganda (17.2%). In each of these countries, they estimated that HIV/AIDS is the leading cause of disease burden (32). When ART was available in the Malawi and Tanzanian sites, and one of the Ugandan sites, we estimate that 25% of deaths occurred in people known to be living with HIV. In the other Ugandan site, Rakai, the estimate was much higher (67%). Historically, HIV incidence and prevalence have been higher in Rakai than Masaka. Since 2005, ART and circumcision services have been available in Rakai, and HIV incidence has fallen. Mortality in Rakai among PLWH and HIV-negative people has been lower than in Masaka, and this difference was especially pronounced before ART was available. This may have been due to research activities in Rakai which included active follow-up for participants who tested HIV-positive, a system of community counselors, mobile clinics which provided sexually transmitted infection treatment and referrals to the static clinics, and nevirapine for pregnant and breastfeeding women with HIV. These activities may have lowered mortality among known PLWH prior to ART treatment being widely available.

Mortality declines have been seen following the introduction of ART. Once treatment programmes were widely available, PLWH mortality fell to about half of the level it was before the introduction of ART. However, PLWH retain a high level of excess mortality with almost 10 deaths to every 1 that would be expected.
